# Early Use of Cryoprecipitate Versus Plasma and Clinical Outcomes in Major Spine Surgery

**DOI:** 10.3390/jcm14207441

**Published:** 2025-10-21

**Authors:** Aparna Depuru, Jong Ok La, Miriam M. Treggiari, Nicole R. Guinn

**Affiliations:** 1 Department of Anesthesiology, Duke University Medical Center, Durham, NC 27710, USA; aparna.depuru@duke.edu (A.D.);; 2 Duke Molecular Physiology Institute, Duke University, Durham, NC 27710, USA

**Keywords:** blood transfusion, cryoprecipitate, plasma, spine surgery, transfusion practices

## Abstract

**Background/Objectives**: Major spine surgery often leads to significant blood loss and coagulopathy, necessitating blood product transfusion. Cryoprecipitate and plasma are two blood products commonly used to manage coagulopathy, but outcomes associated with their use in spine surgery remain unclear. **Methods**: This retrospective cohort study included adult patients undergoing major spine surgery from 2015 to 2022 within a single health system. Included patients received at least one unit of packed red blood cells (PRBCs) followed by either cryoprecipitate or plasma to investigate the treatment of coagulopathy after blood loss. Study endpoints included hospital length of stay (H-LOS), ICU length of stay (ICU-LOS), discharge disposition, one-year mortality, and total blood products transfused up to postoperative day (POD) 2. Multivariable linear and logistic regression was used to estimate associations between the use of cryoprecipitate or plasma and outcomes. **Results**: Of 189 patients meeting inclusion criteria, 120 received cryoprecipitate, and 69 received plasma as the first product after PRBCs. In the univariable analysis, the cryoprecipitate group had lower 1-year mortality (5.0% vs. 14.5%; [95% CI]: 0.31 [0.10, 0.88], *p* = 0.0031) and a shorter ICU-LOS (46 vs. 74 h; [95% CI]: 0.73 [0.53, 1.00], *p* = 0.048). However, despite a trend favoring the cryoprecipitate group, there were no differences between the cryoprecipitate and plasma groups in the multivariable model for H-LOS (adjusted geometric mean ratio [95% CI]: 0.84 [0.68, 1.04], *p* = 0.109), ICU-LOS (adjusted geometric mean ratio [95% CI]: 0.72 [0.50, 1.04], *p* = 0.078), one-year mortality (adjusted OR [95% CI]: 0.49 [0.13, 1.88], *p* = 0.288), or total blood products transfused up to POD2 (adjusted mean difference [95% CI]: −1 unit [−2, 1], *p* = 0.253). Compared with plasma, patients in the cryoprecipitate group were more likely to be discharged to home independently versus disposition to other facility or needing assistance (adjusted OR 0.41 [95% CI]: 0.16, 0.97, *p* = 0.049). **Conclusions**: Use of cryoprecipitate was associated with higher odds of home discharge, while other outcomes were similar between the two groups once adjusting for potential confounders. Further research is needed to better appreciate the clinical impact of the choice of blood products to treat coagulopathy in the setting of bleeding in major spine surgery.

## 1. Introduction

Major spine surgery is often associated with significant blood loss due to the extensive muscle and bone dissection and instrumentation needed for exposure, frequently necessitating transfusion to manage acute anemia [1]. Blood losses and subsequent transfusion of allogeneic red blood cells and/or cell-salvaged blood can cause significant dilution of coagulation factors, which may further contribute to blood losses and ongoing requirement for transfusion of other blood products [2].

While cryoprecipitate and plasma are both used to manage coagulopathy, they differ significantly in volume, composition, and mechanism of action. Plasma is sourced from human donors and contains albumin, coagulation factors, immunoglobulins, and acute-phase proteins. An alternative to plasma to treat coagulopathy related to low fibrinogen is cryoprecipitate, which is derived from plasma and is high in fibrinogen, factor VIII, factor XIII, and von Willebrand factor (VWF) [3]. Transfusion of plasma is associated with potential risk of infection, hemolysis, anaphylaxis, and transfusion-associated circulatory overload [4,5,6]. Cryoprecipitate, which has less volume than plasma, may reduce the risk of transfusion-associated circulatory overload and more promptly restore fibrinogen and has been associated with lower mortality in patients undergoing cardiac surgery [7], whereas transfusion of plasma has been associated with higher mortality in the same population [8]. In spine surgery, use of plasma has been associated with a higher risk of infection and longer hospital length of stay, as well as renal, ischemic, and thrombotic complications [9].

While the adoption of viscoelastic testing (VET) has been associated with a reduction in plasma usage and earlier, more frequent use of cryoprecipitate compared to management guided by conventional laboratory assessments, including in major spine surgery [10], limited research describes outcomes of early use of cryoprecipitate versus plasma in spine surgery. Due to scarce data, coagulopathy management varies widely, driven by provider preference and product availability.

Although plasma and cryoprecipitate have distinct indications, plasma for broad coagulation factor replacement and cryoprecipitate primarily for fibrinogen supplementation, there remains real-world variability in empiric transfusion practices during major spine surgeries. In settings where VET is unavailable, clinicians rely on clinical judgment, with preferences varying between plasma and cryoprecipitate. Additionally, with the potential for rapid blood loss during major spine surgery and the delay in obtaining laboratory results, blood products are often given empirically based on suspicion of clinical coagulopathy. This variation is common, especially in community hospitals or institutions without standardized protocols.

We conducted a cohort study of patients undergoing major spine surgery at a single health system who experienced blood loss requiring packed red blood cell (PRBC) transfusion, followed by at least one unit of either cryoprecipitate or plasma, to compare clinical outcomes based on the first used product for treatment of blood loss-associated coagulopathy.

## 2. Materials and Methods

### 2.1. Study Population

After Institutional Review Board approval (Protocol 00112563, approved 12/2023), we conducted a retrospective review of all adults (age ≥ 18 years) patients undergoing major spine surgery (CPT codes: 22800, 22802, 22804, 22808, 22810, 22812, 22206-8, and 22842-7) between 2015 and 2022 within a single health system (including one academic hospital and two community hospitals). Patients who received at least one unit of PRBCs followed by at least one unit of either cryoprecipitate (given as a pool of 5) or plasma in the operating room (between anesthesia start and anesthesia stop) were included to reflect a population of patients with suspected coagulation abnormalities in the setting of intraoperative bleeding.

### 2.2. Study Exposure and Endpoints

The study exposure was determined based on the first product that was received after at least one unit of the PRBCs. Patients were then assigned to either the cryoprecipitate group or the plasma group. The study endpoints were the length of hospital stay (H-LOS), length of ICU stay (ICU-LOS) among the patients admitted to the ICU, hospital discharge disposition, 1-year mortality, and total blood products transfused after meeting inclusion criteria (excluding any PRBCs before first cryoprecipitate or plasma and first unit of cryoprecipitate or plasma) up to postoperative day (POD) 2 as a short-term bleeding outcome.

### 2.3. Data Collection

Data were extracted from the electronic health record. Variables collected were patient demographics including age; sex; race; height; weight; smoking status; co-morbidities and American Society of Anesthesiologists (ASA) status; preoperative and intraoperative laboratory studies; and procedural information, including hospital location, surgical service line (neurosurgery or orthopedics), case length, use of tranexamic acid, use of cell salvage blood, estimated blood loss (EBL), and total units of PRBCs, platelets, plasma, and cryoprecipitate transfused intraoperatively and postoperatively. A chart review was performed among patients who died to determine the probable cause of death.

### 2.4. Anesthetic and Surgical Management

Restrictive transfusion practices are generally recommended across the health system, including giving single units of PRBCs at a time and using a hemoglobin trigger of 7–8 g/dL in stable, non-hemorrhaging patients. For non-PRBC transfusion, laboratory values generally guide transfusion decisions (fibrinogen < 150–200 mg/dL, platelets < 100 (×10^9^/L), INR > 1.5), although decisions to transfuse are multifactorial, depending on patient condition and left to the individual providers. Use of other blood management techniques, including cell salvage and antifibrinolytics, is requested by the surgeons, generally when anticipated EBL is greater than 1 L. When used, antifibrinolytics were started at the incision. Use of VET is available and encouraged at the university hospital, using ROTEM (Company: Werfen, Bedford, MA, USA) but not available at either of the two community hospitals.

### 2.5. Statistical Analyses

Median (IQR) was used to summarize continuous variables, and counts (percentages) were used for categorical variables. The study endpoints were compared between patients receiving cryoprecipitate as first-line with plasma as first-line. H-LOS and ICU-LOS were log-transformed and analyzed using a multivariable linear regression model. Discharge disposition (home independently vs. a disposition to other facility or needing assistance) and 1-year mortality were analyzed using multivariable logistic regression models. Total blood products transfused after meeting inclusion criteria up through POD2 were analyzed using Wilcoxon rank sum test, and results were reported as mean differences (MDs) with 95% confidence intervals. We first fitted univariable regression models, then we fitted multivariable models adjusting for age, sex, BMI, ASA status, use of tranexamic acid (yes/no), and hospital setting (academic versus community) for each of the study endpoints. Additionally, we conducted a subgroup analysis of patients who received cryoprecipitate versus those who received only plasma. Univariable and multivariable analyses were performed on the subgroup as described for the primary analysis. Results of the log-transformed outcome linear regression model are reported as ratios of geometric means (GMR) with 95% CI, and the results of the logistic regression model are reported as odds ratios (ORs) with 95% CIs. A two-sided alpha level of less than 0.05 was considered statistically significant. All statistical analysis was conducted by the study biostatistician (J.O.L.) using R version 4.4.0.

## 3. Results

### 3.1. Patient Demographic and Baseline Characteristics

Our initial query identified a cohort of 14,256 adult patients who underwent spine surgery. Of those, 585 received PRBCs intraoperatively, and 189 patients met the inclusion criteria, with 120 receiving cryoprecipitate and 69 receiving plasma as a first-line product following the transfusion of at least one unit of PRBCs in the operating room. Table 1 shows the baseline characteristics of the study population. The two groups were comparable for age, race, sex, BMI, co-morbidities, ASA status, and preoperative laboratory values. Hypertension was slightly more common in the cryoprecipitate group, and neurosurgery cases were more frequent, while orthopedic cases predominated in the plasma group.

### 3.2. Intraoperative Blood Product Transfusion Patterns

The total number of intraoperative PRBCs transfused was similar between the two groups, with a median of 3 units (IRQ: 2–5) for the cryoprecipitate group compared to 4 units (IRQ: 2–6) for the plasma group. Use of platelets was also similar (used in 33% of the cryoprecipitate group, median 1 (IQR: 1–2), and 35% of the plasma group, median 2 (IQR: 1–2). Some imbalances were noted in the following transfusion-related variables. Patients managed in the community hospitals were more likely to receive plasma, with only one patient receiving cryoprecipitate as the first-line product. In the cryoprecipitate group, only 17% received subsequent plasma, while a higher percentage of the patients in the plasma group received both plasma and cryoprecipitate (45%).

More patients in the cryoprecipitate group had spine neurosurgery as the primary surgical service (79%), while spine orthopedics was more common in the plasma group (58%). The median case length was slightly longer in the cryoprecipitate group (548 min) compared with the plasma group (498 min), and more patients had posterior versus anterior instrumentation. Compared with the plasma group, the use of cell salvage in the operating room was higher in the cryoprecipitate group (73% vs. 46%), as was the use of tranexamic acid (87% versus 42%) and viscoelastic testing (98% versus 61%).

### 3.3. Study Endpoints

**Figure 1:**
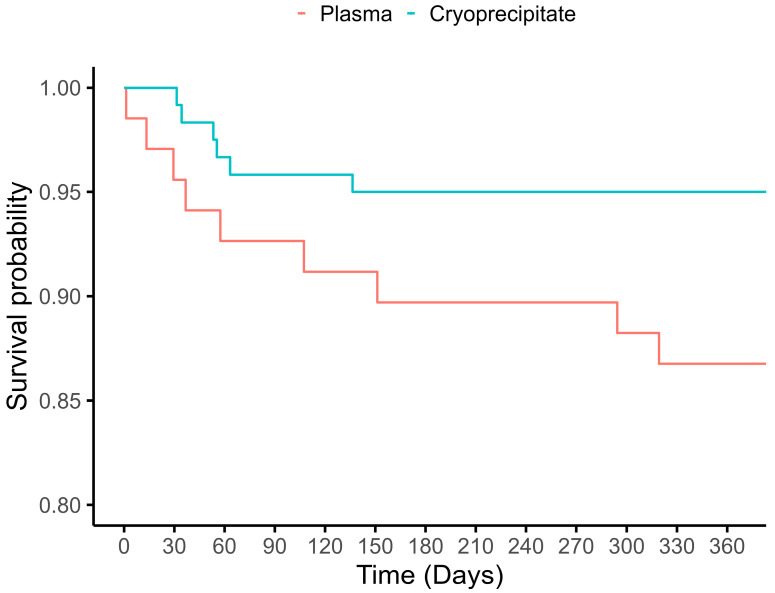
Kaplan–Meier plot showing one-year survival by cryoprecipitate and plasma groups.

Table 2 shows the comparison of clinical outcomes in patients receiving cryoprecipitate versus plasma first. Hospital LOS was similar in the univariable and multivariable analysis between patients in the cryoprecipitate group compared with the plasma group (GMR [95% CI]: 0.84 [0.68, 1.04], *p* = 0.109). The cryoprecipitate group had a shorter ICU LOS among patients admitted to the ICU (46 vs. 74 h; univariable analysis [95% CI]: 0.73 [0.53, 1.00], *p* = 0.048), although this failed to meet statistical significance in the multivariable model (GMR [95% CI]: 0.72 [0.50, 1.04], *p* = 0.078). Likewise, the cryoprecipitate group had lower 1-year mortality in the univariable model (5.0% vs. 14.5%; [95% CI]: 0.31 [0.10, 0.88], *p* = 0.0031, Figure 1), although this was also not significant in the multivariable model (adjusted OR [95% CI]: 0.49 [0.13, 1.88], *p* = 0.288). Discharge disposition was different between groups in the adjusted model, with the patients in the cryoprecipitate group more likely to be discharged home independently compared to being sent to another facility or needing assistance (adjusted OR 0.41 [95% CI]: 0.16, 0.97, *p* = 0.049).

As a surrogate of short-term bleeding, total blood products transfused (PRBCs, platelets, plasma, and cryoprecipitate) from the time of meeting the inclusion criteria through POD2 were evaluated. The median number of total units transfused was slightly higher in the plasma group [5 units; IQR: 3–9] compared to the cryoprecipitate group [4 units; IQR: 2–8], but this difference did not reach statistical significance (mean difference [95% CI]: −1 unit [−2, 1], *p* = 0.253).

The subgroup analysis of patients receiving only cryoprecipitate (N = 100) versus only plasma (N = 38) had similar results to the primary analysis (Table 3), with a trend favoring the cryoprecipitate group, which was only significant for discharge disposition (adjusted OR 0.16, 95% CI: 0.03, 0.69, *p* = 0.025).

Infection was the most common cause of death by one year, accounting for a higher proportion in the plasma group (Table 4).

## 4. Discussion

Our study found that in a cohort of patients undergoing major spine surgery, approximately one-third of patients who received PRBCs intraoperatively were subsequently administered either cryoprecipitate or plasma, with cryoprecipitate being more commonly administered. The two groups were similar in terms of demographics, although cryoprecipitate was less commonly used at the community hospitals than at the university hospital. Although all clinical outcomes favored the cryoprecipitate group, only ICU-LOS and mortality achieved significance in the univariable model, while only discharge disposition achieved significance in the multivariable model.

This is the first study to analyze early cryoprecipitate and plasma use in spine surgery and their perioperative outcomes. Our study findings of variation of cryoprecipitate and plasma use among providers are consistent with research in other surgical settings [11]. In our study, patients who received cryoprecipitate first were more likely to be in the neurosurgical service, whereas patients who received plasma transfusion were more likely to be in the orthopedic service. Although this may reflect differing practice patterns, it may also relate to hospital location, with more neurosurgical spine surgeons at the main academic center versus more orthopedic procedures in the community hospitals. The higher use of cryoprecipitate at the university hospital may reflect the provider’s preference or product availability. Given these local differences, our findings may not have fully accounted for patient clustering within hospitals, despite adjusting for location, and this may have affected the difference seen in postoperative disposition.

We also evaluated a short-term bleeding outcome of total blood products transfused from meeting study inclusion to POD2 as a proxy for perioperative bleeding severity. This metric did not differ significantly between groups, suggesting that early cryoprecipitate use may not decrease total transfusion burden. To address bleeding in the surgical setting, there is little evidence to support distinct clinical or laboratory-based triggers for plasma and cryoprecipitate transfusion [12]. Hypofibrinogenemia has been associated with increased blood loss and transfusion in spine surgery [13], and guidelines suggest transfusing at a plasma fibrinogen level of less than 1.5–2 g/L in bleeding patients [14], although there is no precise threshold point for clinically significant hypofibrinogenemia [14]. Use of VET has favored the use of cryoprecipitate [10], and in our study, more patients in the cryoprecipitate group (97.5%) had VET performed in comparison to the plasma group (60.9%). Although traditional laboratory studies, including INR, are an alternative to VET for assessing the degree of coagulopathy, the time required for results in patients with acute intraoperative blood loss can deter many clinicians from conducting these studies, favoring empiric treatment of suspected clinical coagulopathy. In our study, only a minority of patients (26.5%) had INR checked prior to transfusion of plasma or cryoprecipitate.

Despite the need to manage bleeding and coagulopathy, blood component transfusion is associated with complications such as anaphylaxis, transfusion-related acute lung injury, transfusion-associated circulatory overload, and transfusion-related immunomodulation [14]. Interestingly, in our study, of those who died within one year, infection was the most common cause of death, with the survival curves separating between the two groups early after the surgery (Figure 1). Given the limited sample size, we cannot draw definitive conclusions; however, the higher proportion of infection-related deaths in the plasma group may reflect the relatively increased immunosuppression risk associated with plasma transfusion, among other contributing factors.

This study has several limitations. In addition to being a retrospective, single-health-system study, we were unable to examine how abnormal laboratory studies influenced decisions of which product to transfuse. Cryoprecipitate and plasma differ in both their components and their indications for transfusion, and the decision to choose one product over the other is multifactorial, including product availability, use of conventional laboratory testing and/or VET, and personal preference. It is also worth noting that in terms of the dose of plasma, many of the patients can be considered to be underdosed. The standard dose of plasma is 15–20 mL/kg, yet in this study, 42% of the patients in the plasma group received only a single unit of plasma. An additional limitation of our study is the potential for time-to-transfusion bias. While most hospitals maintain thawed or liquid plasma for immediate availability, cryoprecipitate typically requires on-demand thawing, which can delay administration by 30 to 45 min even under optimal conditions. This delay may have influenced the timing of transfusion decisions and introduced unmeasured confounding related to patient stability or clinical urgency. Therefore, we cannot ensure that indication bias and practice variability were fully accounted for in our analyses and note that these findings may not generalize to other settings. During the study period, there were several brief periods of cryoprecipitate shortage, during which the providers were instructed to use plasma preferentially. Although time-limited, these shortages introduced some real-world variability in blood product assignment independent of provider choice and potentially mitigated the residual confounding. However, given the higher likelihood of receiving cryoprecipitate at the university hospital, it is possible that our adjustments in the multivariable models did not allow us to completely account for bias or residual confounding, despite adjusting for hospital location. Therefore, our results should be interpreted as hypothesis-generating, and future prospective design studies should be performed to test these hypotheses.

In this study, we did not examine the use of fibrinogen factor concentrates or prothrombin complex concentrates. Although these have been studied for bleeding-related coagulopathy in major surgery, they are rarely used in our operative setting due to limited availability and the need for hematology approval. The cost and limited availability, along with limited clinical evidence supporting the effectiveness of clotting factor preparations in surgery, have resulted in a lack of standardization of coagulation factor concentrates relative to the blood product transfusion [9].

## 5. Conclusions

There is limited research on blood product administration to manage coagulopathy during major spine surgery, a procedure often requiring transfusions due to significant blood loss. In our study, patients receiving cryoprecipitate versus plasma as a first-line product were more likely to be discharged home and had a lower 1-year mortality and shorter ICU-LOS, although the differences in mortality and ICU-LOS did not retain significance after accounting for measured confounders. Given the lower cost of plasma to cryoprecipitate, if clinical outcomes are similar, plasma may be preferable. If early cryoprecipitate truly improves outcomes and this study was underpowered or missed confounders, its higher cost may be justified as a first-line treatment for coagulopathy in spine surgery. Further prospective research is needed to guide transfusion practices for managing coagulopathy in this population.

## Figures and Tables

**Table 1:** Patient demographics, laboratory values, and surgical and intraoperative transfusion characteristics. Values are expressed as numbers (%) or medians [interquartile ranges] unless otherwise specified.

	Overall (n = 189)	Cryoprecipitate (n = 120)	Plasma (n = 69)
Age, years	68.0 [59.0, 73.0]	67.0 [60.0, 73.0]	69.0 [58.0, 73.0]
Race, n (%)			
Asian	2 (1.1%)	0 (0%)	2 (2.9%)
Black or African American	28 (14.8%)	19 (15.8%)	9 (13.0%)
Caucasian/White	154 (81.5%)	97 (80.8%)	57 (82.6%)
Other	5 (2.6%)	4 (3.3%)	1 (1.4%)
Female, n (%)	129 (68.3%)	83 (69.2%)	46 (66.7%)
Weight, kg	77.4 [64.4, 90.7]	76.6 [64.3, 88.8]	78.3 [67.1, 91.5]
BMI	27.5 [23.9, 32.4]	27.6 [24.1, 32.9]	27.5 [23.7, 31.4]
Diabetes, n (%)	23 (12.2%)	14 (11.7%)	9 (13.0%)
Hypertension, n (%)	83 (43.9%)	57 (47.5%)	26 (37.7%)
Smoking status, n (%)	7 (3.7%)	4 (3.3%)	3 (4.3%)
ASA status, n (%)			
1 or 2	35 (18.5%)	22 (18.3%)	13 (18.8%)
3 or 4	154 (81.5%)	98 (81.7%)	56 (81.2%)
Preoperative Laboratory studies *			
Hemoglobin, g/dL	12.4 [11.3, 13.5]	12.5 [11.7, 13.5]	12.0 [10.7, 13.8]
Platelet count (×10^9^/L)	237 [198, 278]	244 [201, 279]	227 [190, 274]
INR	1.00 [1.00, 1.10]	1.00 [1.00, 1.10]	1.10 [1.00, 1.15]
Emergency Status, n (%)	2 (1.1%)	1 (0.8%)	1 (1.4%)
Surgical Service, n (%)			
Neurosurgery	124 (65.6%)	95 (79.2%)	29 (42.0%)
Orthopedics	65 (34.4%)	25 (20.8%)	40 (58.0%)
Location, n (%)			
University Hospital	162 (85.7%)	119 (99.2%)	43 (62.3%)
Community Hospital	27 (14.3%)	1 (0.8%)	26 (37.7%)
Case length, minutes	528 [451, 639]	548 [472, 632]	498 [395, 644]
Procedure by CPT codes **			
Anterior arthrodesis/instrumentation 2–3 levels (22808, 22845)	5 (2.6%)	1 (0.8%)	4 (5.8%)
Anterior arthrodesis/instrumentation 4–7 levels (22810, 22846)	4 (2.1%)	2 (1.7%)	2 (2.9%)
Posterior arthrodesis/instrumentation up to 6 levels (22800, 22842)	67 (35.4%)	33 (27.5%)	34 (49.3%)
Posterior arthrodesis/instrumentation 7–12 levels (22802, 22843)	151 (79.9%)	116 (96.7%)	35 (50.7%)
Posterior arthrodesis/instrumentation 13+ levels (22804, 22844)	46 (24.3%)	30 (25%)	16 (23.2%)
Posterior or lateral osteotomy (22206, 22207, 22208)	47 (24.9%)	36 (30%)	11 (15.9%)
PRBCs, n (%)	189 (100%)	120 (100%)	69 (100%)
N of PRBC units	4 [2, 5]	3 [2, 5]	4 [2, 6]
Cryoprecipitate, n (%)	151 (79.9%)	120 (100%)	31 (44.9%)
No. of Cryoprecipitate units	2 [1, 2]	2 [1, 2]	1 [1, 2]
Plasma, No. (%)	89 (47.1%)	20 (16.7%)	69 (100%)
No. of Plasma units	2 [1, 3]	2 [1, 2.3]	2 [1, 3]
Plasma and Cryoprecipitate, n (%)	51 (27.0%)	20 (16.7%)	31 (44.9%)
Apheresis platelet, n (%)	64 (33.9%)	40 (33.3%)	24 (34.8%)
No. of Apheresis platelet units	1 [1, 2]	1 [1, 2]	2 [1, 2]
Cell saver intake, n (%)	120 (63.5%)	88 (73.3%)	32 (46.4%)
Cell saver volume, mL	581 [375, 932]	572 [377, 932]	652 [375, 914]
Estimated Blood Loss (mL)	2300 [1500, 3650]	2400 [1500, 3700]	2050 [1500, 3500]
Tranexamic Acid, n (%)	133 (70.4%)	104 (86.7%)	29 (42.0%)
Total dose, g	3.41 [1.97, 4.75]	3.70 [2.45, 4.82]	2.45 [1.42, 4.22]
Intraoperative ROTEM, n (%)	159 (84.1%)	117 (97.5%)	42 (60.9%)
First FIBTEM A10	15.0 [12.0, 18.0]	15.0 [12.0, 18.0]	15.0 [12.0, 19.8]
Lowest FIBTEM A10	10.0 [8.0, 12.0]	10.0 [8.0, 12.0]	10.0 [6.0, 13.5]
First EXTEM A10	57.0 [51.0, 61.5]	57.0 [51.0, 61.0]	56.5 [52.3, 63.0]
Lowest EXTEM A10	48.0 [42.0, 52.0]	48.0 [43.0, 51.0]	46.0 [39.0, 53.8]
EXTEM Clotting Time	62.0 [56.0, 68.0]	62.0 [55.0, 66.0]	67.0 [59.0, 74.8]
Highest EXTEM CT	69.0 [64.0, 77.0]	68.0 [62.0, 74.0]	74.5 [69.0, 85.0]

ASA: American Society of Anesthesiologists; BMI: Body Mass Index; CPT: current procedural terminology; ROTEM: rotational thromboelastometry; plasma; INR: international normalized ratio; PRBC: packed red blood cell. * Preoperative laboratory studies missing-hemoglobin and platelet count: 19 in cryoprecipitate group and 14 in plasma group, INR: 97 in cryoprecipitate group and 42 in plasma group. ** Patient may have more than 1 CPT code; totals add to more than 100%.

**Table 2:** Clinical outcomes in patients receiving cryoprecipitate versus plasma as a first-line agent—univariable and multivariable models.

					Univariable		Multivariable	
Endpoints	N_Cryo_	Cryoprecipitate	N_plasma_	Plasma	Estimate (95% CI)	*p*	Adjusted Estimate (95% CI)	*p*
Total blood products transfused	105	4 [2, 8]	65	5 [3, 9]	−1 [−2, 1]	0.253	-	-
Hospital LOS (days) *	120	7.6 [5.5, 10.5]	69	7.4 [5.5, 12.5]	0.96 (0.80, 1.15)	0.648	0.84 (0.68, 1.04)	0.109
ICU-LOS (hours) *	96	46.3 [27.8, 95.7]	49	74.4 [36.3, 157]	0.73 (0.53, 1.00)	0.048	0.72 (0.50, 1.04)	0.078
Discharge Disposition **	120		67					
Home		49 (40.8%)		22 (32.8%)	ref	-	ref	-
Home with assistance/long-term facility		71 (59.2%)		45 (67.2%)	0.71 (0.37, 1.32)	0.281	0.41 (0.16, 0.97)	0.049
1-year Mortality **	120		69					
Alive		114 (95.0%)		59 (85.5%)	ref	-	ref	-
Dead		6 (5.0%)		10 (14.5%)	0.31 (0.10, 0.88)	0.031	0.49 (0.13, 1.88)	0.288

LOS: length of stay; ICU: intensive care unit; Median [Q1, Q3] for total blood products transfused, H-LOS and ICU-LOS and N (%) for discharge disposition and 1-year mortality. Reference group is patients who received plasma as first-line treatment. Total blood products given from intraoperative after the first cryoprecipitate or plasma to POD2 (by calendar date). Wilcoxon rank sum test for total blood products transfused. Linear regression for log-transformed H-LOS and ICU-LOS. Logistic regression for 1-year mortality and discharge disposition. Estimates reporting * ratio of geometric means; ** odds ratio. Multivariable regression includes adjustment for age, sex, BMI, ASA status, anti-fibrinolytic use, and hospital stay.

**Table 3:** Clinical outcomes in subgroup analysis of patients receiving only cryoprecipitate versus only plasma for management of coagulopathy.

					Univariable		Multivariable	
Endpoints	N_Cryo_	Cryoprecipitate	N_plasma_	Plasma	Estimate (95% CI)	*p*	Adjusted Estimate (95% CI)	*p*
Total blood products transfused	85	4 [2, 7]	34	3 [2, 5]	1 [−0.5, 2]	0.378	-	-
Hospital LOS (days) *	100	7.6 [5.5, 10.5]	38	6.5 [4.6, 8.3]	1.09 (0.88, 1.35)	0.415	0.87 (0.64, 1.19)	0.388
ICU-LOS (hours) *	80	44.9 [26.1, 99.0]	19	68.2 [39.4, 126]	0.86 (0.56, 1.30)	0.465	0.83 (0.46 1.49)	0.530
Discharge Disposition **	100		37					
Home		39 (39.0%)		12 (32.4%)	ref	-	ref	-
Home with assistance/long-term facility		61 (61.0%)		25 (67.6%)	0.75 (0.33, 1.64)	0.481	0.16 (0.03, 0.69)	0.025
1-year Mortality **	100		38					
Alive		94 (94.0%)		32 (84.2%)	ref	-	ref	-
Dead		6 (6.0%)		6 (15.8%)	0.34 (0.10, 1.16)	0.079	0.52 (0.10, 3.22)	0.447

LOS: length of stay; ICU: intensive care unit; Median [Q1, Q3] for total blood products transfused, H-LOS and ICU-LOS and N (%) for discharge disposition and 1-year mortality. Reference group is patients who received plasma as first-line treatment. Total blood products given from intraoperative after the first cryoprecipitate or plasma to POD2 (by calendar date). Wilcoxon rank sum test for total blood products transfused. Linear regression for log-transformed H-LOS and ICU-LOS. Logistic regression for 1-year mortality and discharge disposition. Estimates reporting * ratio of geometric means; ** odds ratio. Multivariable regression includes adjustment for age, sex, BMI, ASA status, anti-fibrinolytic use, and hospital stay.

**Table 4:** One-year mortality and causes of death.

	Overall (n = 189)	Cryoprecipitate (n = 120)	Plasma (n = 69)
1-year mortality	16 (8.5%)	6 (5.0%)	10 (14.5%)
Causes of death			
Infection/sepsis	8 (4.2%)	3 (2.5%)	5 (7.2%)
Cancer	5 (2.6%)	3 (2.5%)	2 (2.9%)
Gastrointestinal bleed	1 (0.5%)	0 (0%)	1 (1.4%)
Unknown	2 (1.1%)	0 (0%)	2 (2.9%)

## Data Availability

Data is not available due to a lack of a data-sharing agreement.
